# Supplementary material for: Gluconate-Lactobionate-Dextran Perfusion Solutions Attenuate Ischemic Injury and Improve Function in a Murine Cardiac Transplant Model
Source: Cells. 2022 May 16;11(10):1653. doi: 10.3390/cells11101653 (PMC9139252; doi:10.3390/cells11101653)
Supplement: Supplementary file 1 [file cells-11-01653-s001.zip › cells-1700749-supplementary.pdf]

HTK

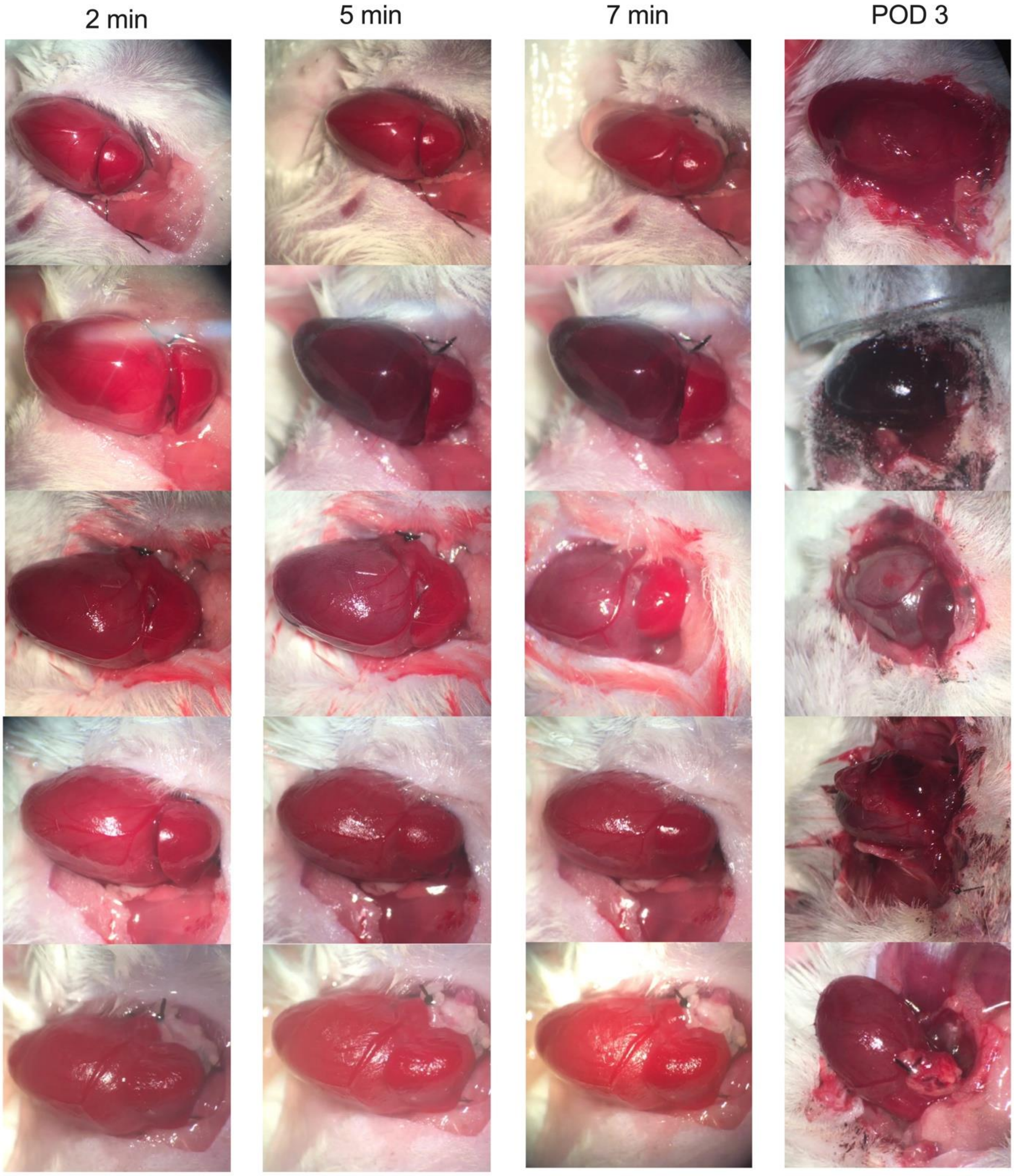

Supplementary Figure S1. Representative images of cardiac grafts perfused and stored in HTK at two, five and seven minutes after reperfusion as well as on POD 3 before biopsy sampling.

UHK

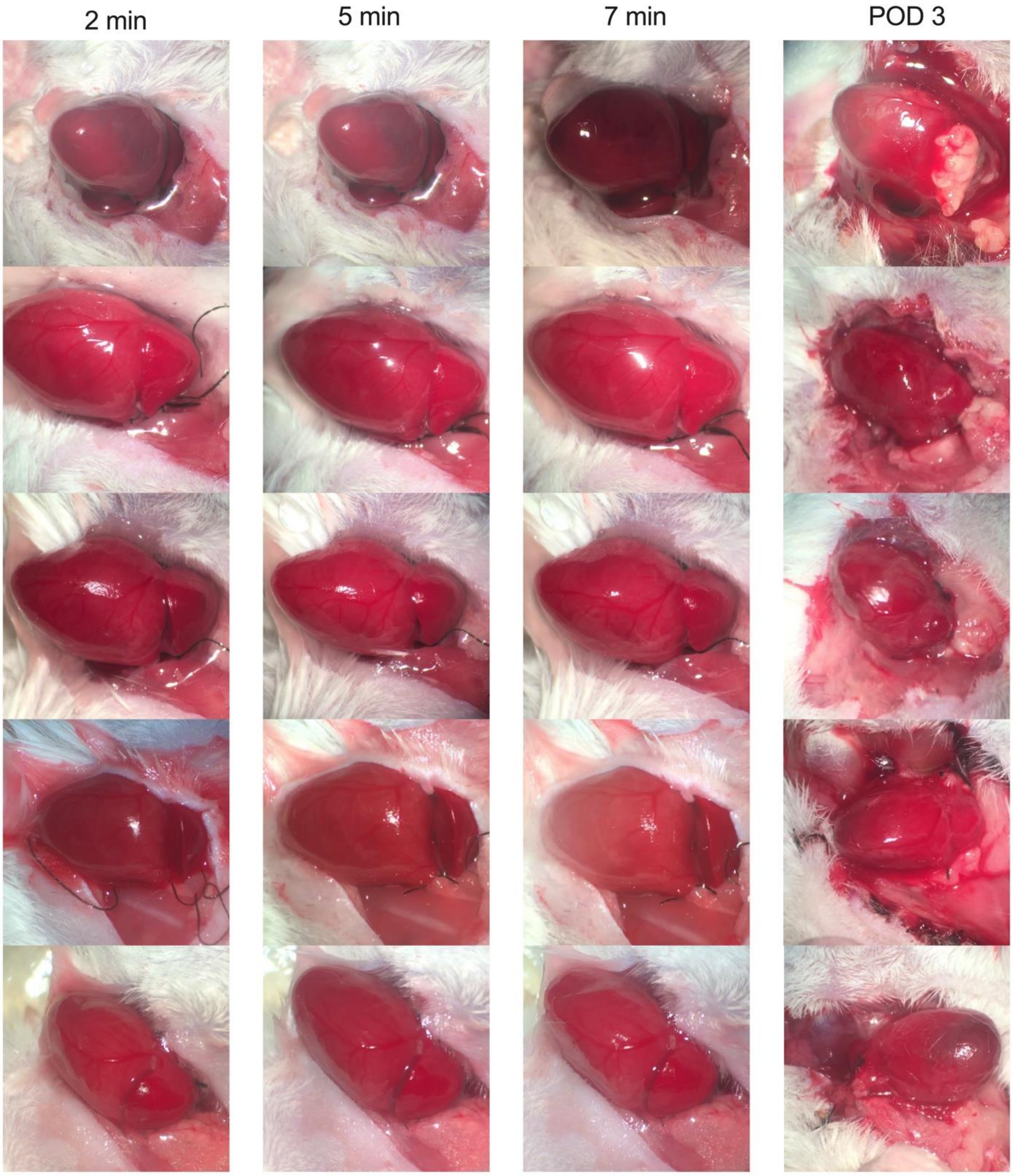

Supplementary Figure S2. Representative images of cardiac grafts perfused and stored in UHK at two, five and seven minutes after reperfusion as well as on POD 3 before biopsy sampling.

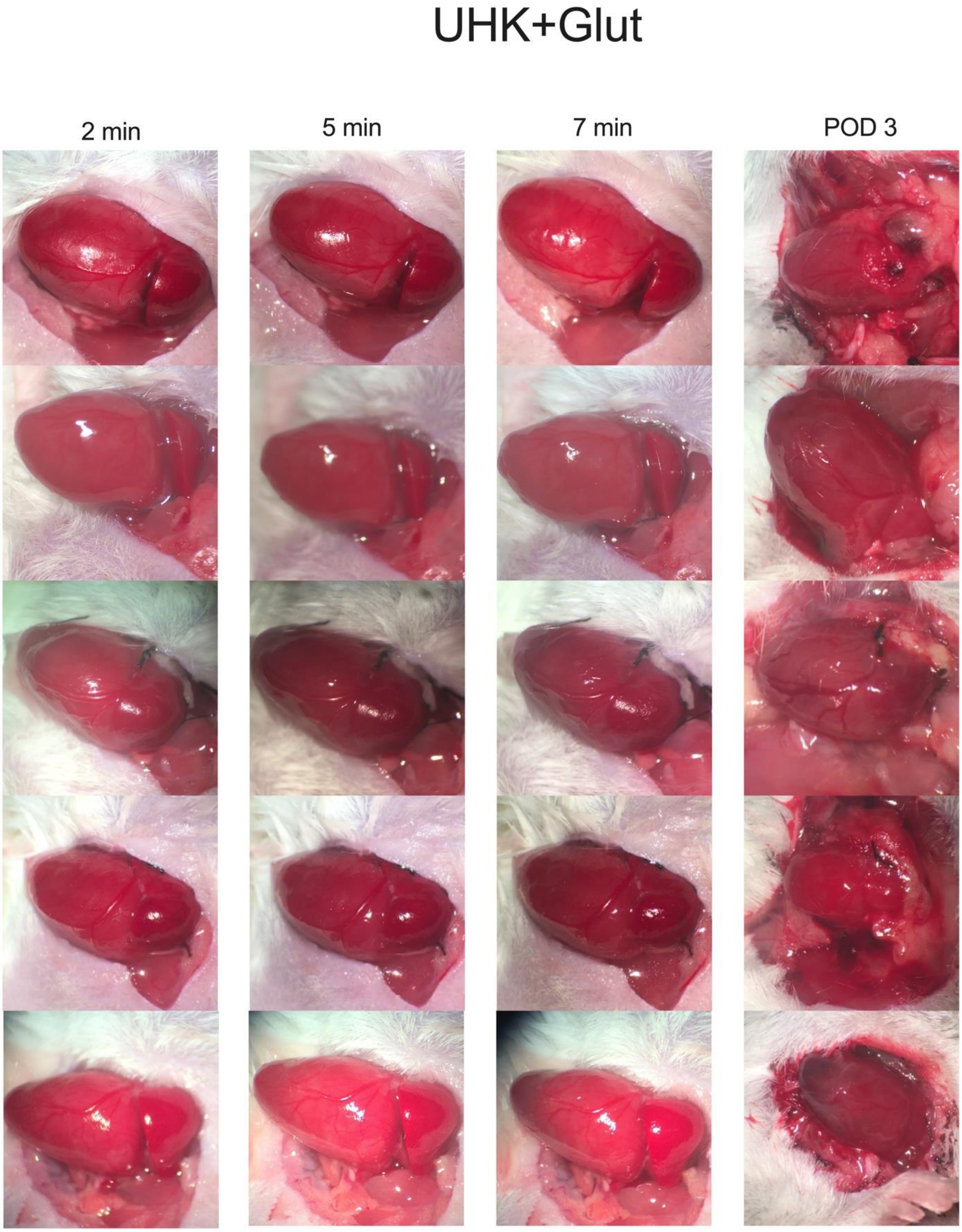

Supplementary Figure S3. Representative images of cardiac grafts perfused and stored in UHK + Glut at two, five and seven minutes after reperfusion as well as on POD 3 before biopsy sampling.

ULK

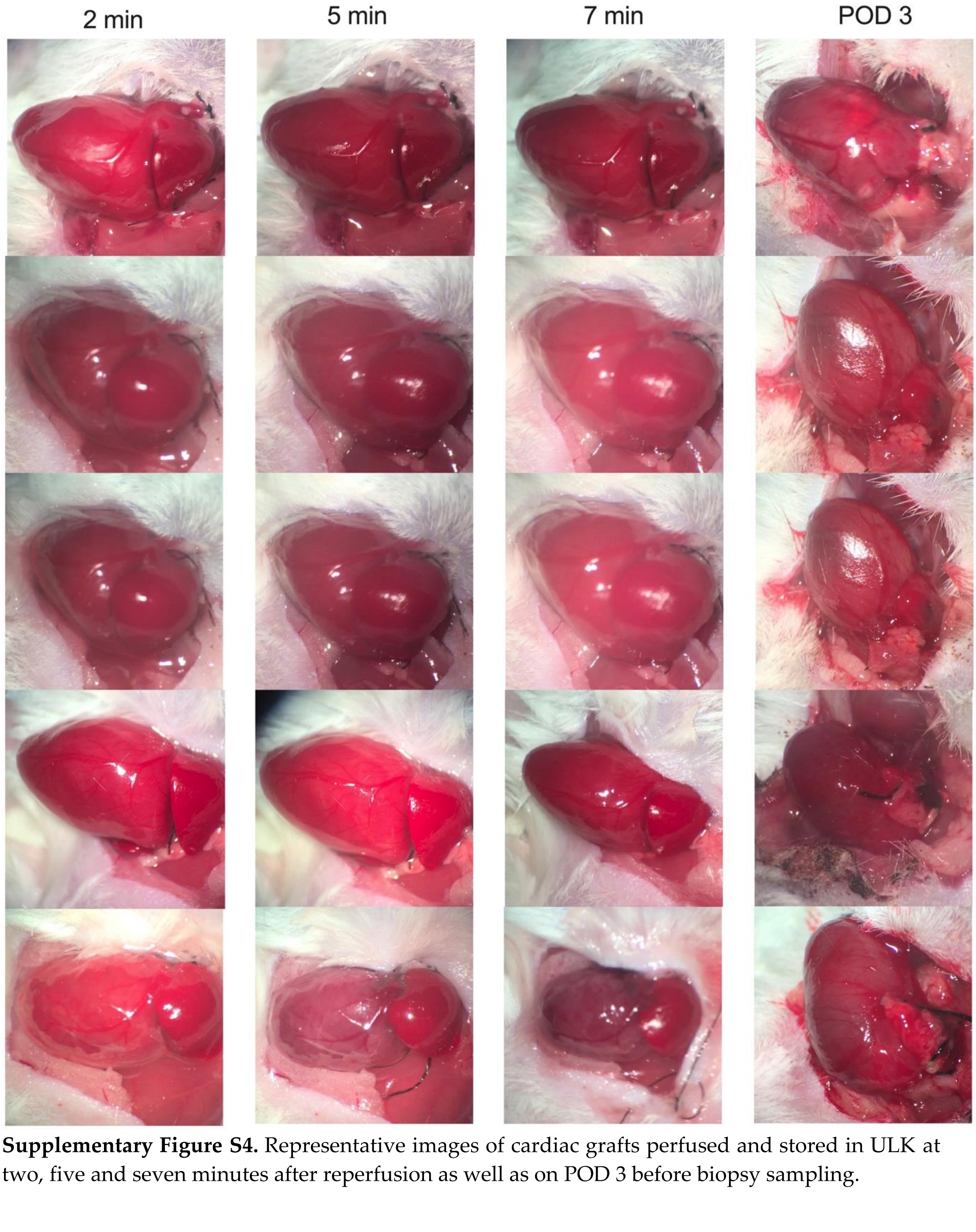

Supplementary Figure S4. Representative images of cardiac grafts perfused and stored in ULK at two, five and seven minutes after reperfusion as well as on POD 3 before biopsy sampling.

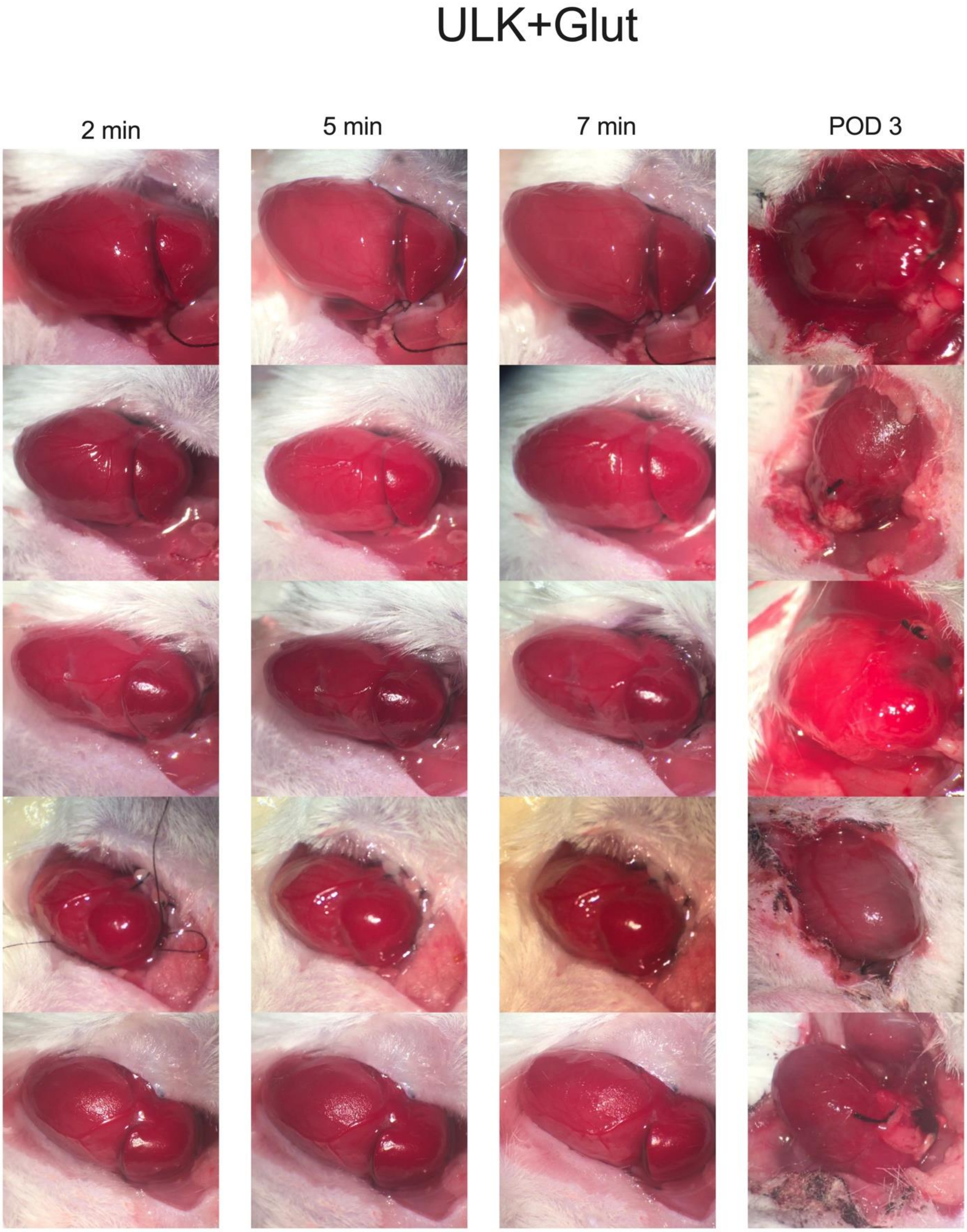

Supplementary Figure S5. Representative images of cardiac grafts perfused and stored in ULK + Glut at two, five and seven minutes after reperfusion as well as on POD 3 before biopsy sampling.
